# Targeting Modified Lipids during Routine Lipidomics Analysis using HILIC and C30 Reverse Phase Liquid Chromatography coupled to Mass Spectrometry

**DOI:** 10.1038/s41598-019-41556-9

**Published:** 2019-03-25

**Authors:** Thu Huong Pham, Muhammad Zaeem, Tiffany A. Fillier, Muhammad Nadeem, Natalia P. Vidal, Charles Manful, Sukhinder Cheema, Mumtaz Cheema, Raymond H. Thomas

**Affiliations:** 1 School of Science and the Environment/Boreal Ecosystem Research Initiative, Grenfell Campus, Memorial University of Newfoundland, Corner Brook, Newfoundland, A2H 5G4 Canada; 2 Department of Biochemistry, Memorial University of Newfoundland, St John’s, Newfoundland, A1B 3x9 Canada; 3 Department of Environmental Sciences, COMSATS University of Islamabad, Vehari, 61100 Pakistan

## Abstract

Lipids are important biomolecules in all biological systems and serve numerous essential cellular functions. The global analysis of complex lipids is very challenging due to the extreme diversity in lipid structures. Variation in linkages and positions of fatty acyl chain(s) on the lipid backbone, functional group modification, occurrence of the molecular species as isomers or isobars are among some of the greatest challenges to resolve in lipidomics. In this work, we describe a routine analytical approach combining two liquid chromatography platforms: hydrophilic interaction (HILIC) and C30 reversed-phase chromatography (C30RP) coupled to high resolution mass spectrometry (HRMS) as complementary high throughput platforms to analyze complex lipid mixtures. Vascular plants (kale leaves and corn roots), rat brain and soil microbes were used as proxies to evaluate the efficiency of the enhanced approach to resolve traditional, as well as, modified lipids during routine lipidomics analysis. We report for the first time, the observation of a modified class of acylphosphatidylglycerol (acylPG) in corn roots by HILIC, and further resolution of the isomers using C30RP chromatography. We also used this approach to demonstrate the presence of high levels of *N*-monomethyl phosphatidylethanolamine (MMPE) in soil microbes, as well as to determine the regioisomers of lysophospholipids in kale leaves. Additionally, neutral lipids were demonstrated using C30RP chromatography in positive ion mode to resolve triacylglycerol isomers in rat brain. The work presented here demonstrates how the enhanced approach can more routinely permit novel biomarker discovery, or lipid metabolism in a wide range of biological samples.

## Introduction

Lipidomics is the omics science concerned with the comprehensive identification and quantification of cellular lipid molecular species and their function in biological systems. Improvement in chromatography and mass spectrometry have contributed significantly to the development and rapid expansion of lipidomics as a sub discipline in metabolomics over recent years^1,2^. The high mass resolution and mass accuracy integrated in advanced mass spectrometers provides accurate measurement of the mass of precursor ions, which enables isobaric separation, as well as fragment ions to eliminate possible false positive identification^3^. Lipids comprise an essential part of all biological systems, and serve numerous structural and functional roles in these systems inclusive of providing structural molecules for forming cellular membrane bilayers^4^, act as signaling molecules in cell communications (*e*.*g*., diacylglycerols and ceramides)^5,6^, energy storage (*e*.*g*., triacylglycerols in adipose tissues) and transport^7^. Lipids can be classified mainly into eight categories, namely, fatty acids (FA), glycerolipids (GL), glycerophospholipids (GP), sphingolipids (SP), sterol lipids (ST), prenol lipids (PR), saccharolipids (SL) and polyketides (PK)^8,9^. Separation and identification of modified lipids in the lipidome of biological systems are of considerable interest considering their significant roles in normal and aberrant cellular lipid metabolism^10,11^. Modifications can occur on the phospholipid headgroups or with the linkages joining the lipid monomers in complex lipid classes. As an example, fatty acids can be esterified to the glycerol headgroup of phosphatidylglycerol to form acylphosphatidylglycerol (acylPG) which is typically found in the membrane of some prokaryotic species^12^. Phosphatidylglycerol (PG) is a major phospholipid in chloroplasts and mitochondria, which are well-known for their association with plant sensitivity to cold temperature^13^. The unsaturation degree and the position of fatty acyl chains on the glycerol backbone of PG also have implications in biosynthesis and environmental stress responses^13,14^. For example, C16 fatty acyl chain at *sn*-2 position of PGs indicates the molecules were made in the chloroplast, whereas the appearance of C18 fatty acyl chain at *sn-*2 in PGs reveals the non-chloroplast source of PG, such as PG made in the endoplasmic reticulum (E.R.)^14^. Thus, determining the position of the fatty acyl chains in PGs may have significance in studying the pathway of synthesis during environmental stress response or normal development. Another important type of lipid modification involves the linkages at the *sn*-1 position of the glycerol moiety leading to the formation of ether and vinyl ether (i.e. plasmalogen) lipids. Identification of these small alternations in lipid molecular structures provide significant challenges during routine lipidomic analysis for a wide range of biological samples^15,16^.

The use of high-performance liquid chromatography (HPLC) is a well-recognized approach in the lipidomics field for routine analysis of complex lipid mixtures, consisting of either normal phase (NPLC) or reverse-phase liquid chromatography (RPLC). The former typically separates lipids based on their polar functional group, while the latter resolves lipid species based on their lipophilic composition. Currently, there is an interest in the use of hydrophilic interaction liquid chromatography (HILIC) which enables separation of lipid classes based on headgroup composition, similar to that observed with NPLC; while using the same RPLC mobile phases to improve ionization efficiency and reproducibility^17,18^. HILIC can be used as an alternate or complementary system in a mix-mode with reverse phase liquid chromatography in the analysis of complex lipids^18–20^.

In contrast to the separation of lipids in HILIC, RPLC is governed by the interaction between the hydrophobic stationary phase and the hydrophobicity of the fatty acyl chains. This allows intra-class lipid separation or differentiation of lipid based on their carbon chain length and degree of unsaturation. Although there are many non-polar stationary phases used in lipid analysis, the most widely used are stationary phases consisting of octadecylsilyl (C18) groups^21–23^. Recently, the use of C30 stationary phase has been shown to potentially be a promising high throughput untargeted lipidomics platform for analyzing complex biological samples^24,25^. Specifically, C30 reverse phase chromatography was shown to offer higher shape selectivity permitting superior resolution of geometric lipid isomers compared to C18 reverse phase chromatography^26,27^. Applications of C30 reverse phase chromatography specifically for the resolution of neutral lipid (triglycerides) isomers in edible oils were also reported^28^. It was observed that a C30 column allowed excellent resolution of triglyceride regioisomers, which only differed by the alternation of fatty acid positions (*sn*-1, 2 or 3) on the glycerol backbone^28^. Regioisomerism has been a target in the development of analytical methods using mass spectrometry, such as collision-induced dissociation (CID) in tandem time-of-flight (i.e.., ToF-ToF) mass spectrometers^29,30^, multistage mass spectrometry (i.e., MS^n^, where n > 3) in a linear ion trap^31,32^, or incorporating the ozonolysis in an ion-molecule reaction ion trap (i.e., CID-OzID technique)^33,34^, to name a few. Taking these into consideration, we sought to apply HILIC complementary with C30 reverse phase chromatography coupled with high resolution tandem mass spectrometry (HILIC/C30RPLC-HRMS/MS) as a high throughput lipidomics platform to routinely analyze complex lipids (isobars, regioisomers, ether-linkage or headgroup modification) in diverse biological systems.

## Experimental Section

### Chemicals

HPLC grade acetonitrile, chloroform and methanol were purchased from Fisher Scientific, Canada; deionized water was obtained from PURELAB Purification System (ELGA Labwater, ON, Canada). HPLC grade acetic acid, formic acid, ammonium formate and ammonium acetate were purchased from Sigma-Aldrich (ON, Canada). Each lipid class was purchased as individual from Avanti Polar Lipids (Alabaster, AL, USA) and used to make a complex lipid standard mixture consisting SQDG 16:0/16:0, MGDG 16:3(7Z,10Z,13Z)/18:3(9Z,12Z,15Z), PA 18:1(9Z)/18:1(9Z), PG 18:0/20:4(5Z,8Z,11Z,14Z), PC 18:0/20:4(5Z,8Z,11Z,14Z), PE 18:0/20:4(5Z,8Z,11Z,14Z), diether PC O-18:0/O-18:0, plasmalogen PC P-18:0/20:4(5Z,8Z,11Z,14Z), plasmalogen PE P-18:0/20:4(5Z,8Z,11Z,14Z), MMPE 16:0/16:0 and DMPE 16:0/16:0, LPA 20:4(5Z,8Z,11Z,14Z), LPC 18:1(9Z), LPE 18:0, plasmalogen LPE P-18:0; PI 18:0/20:4(5Z,8Z,11Z,14Z); DLCL 18:2(9Z,12Z)/18:2(9Z,12Z), and SM d18:1/18:0. Aliquots of the prepared standard mixture were used for method development.

### Lipid extraction

Biological samples representing vascular plants (kale leaves and corn roots), animal (rat brain) and soil microbes were used as proxies to evaluate the efficiency of the high throughput lipidomics platform for the analysis of complex lipid classes (*e*.g., modified lipids) in biological samples. Corn roots and podzolic soil samples were obtained following field cultivation under cool climatic conditions at Pynn’s Brook Research Station, Pasadena, Newfoundland, Canada. Kale plants cultivated under greenhouse conditions using natural media amendments were provided by Dr. Lord Abbey^35^. Rat brain samples were provided by Dr. Sukhinder Cheema (Biochemistry, St. John’s, Memorial University of Newfoundland). Ethics approval for this study was granted by Memorial University Animal Care Committee as mandated by the Canadian Council on Animal Care and all the experiments were performed in accordance with relevant guidelines and regulations. Total lipids from each sample were extracted using the Bilgh and Dyer method. All plant samples were incubated by immersion in hot isopropanol for 10 minutes prior to extraction. Samples were cryo-homogenized (Cryomill, Retsch, Germany) and 10–100 mg of homogenized sample (4 replications for each sample type) transferred to glass centrifuge tubes to which 1 mL methanol containing 0.01% butylated hydroxytoluene and 1 mL of chloroform was added. The sample mixture was vortexed, then added 0.8 mL of water and centrifuged at 5,000 × g for 15 min. Following centrifugation, the organic (bottom) layer containing the lipids was transferred to a pre-weighed 2 mL sample vial having a PTFE lined cap (VWR). The sample was dried under a stream of nitrogen, and the vial re-weighed to determine the amount of lipid recovered^36^. The recovered sample was then re-suspended in 1 mL chloroform:methanol (1:1 v/v) and stored at −20 °C for analysis by liquid chromatography coupled with high resolution tandem mass spectrometry.

### Analysis of the lipid mixture using HILIC or C30-RPLC

The HILIC column (Luna 3 µm, 100 × 2 mm I.D., particle size: 3 µm, pore diameter: 200 Å) was purchased from Phenomenex (Torrance, CA, USA). The mobile phase system used was as follows: acetonitrile:water (97:3 v/v) containing 10 mM ammonium acetate (solvent A), and solvent B containing 10 mM ammonium acetate in pure water. HILIC separation was carried out at 25 °C (column oven temperature) with a flow rate of 0.2 mL/min, and 10 µL of the lipid extract suspended in chloroform:methanol (1:1 v/v) injected in the machine. The system gradient was maintained at 100% solvent A for 2 min; solvent B was then increased to 10% over 23 min, solvent B was increased from 10–15% over 10 min, and then kept at 15% B for 5 min. The column was re-equilibrated to starting conditions (100% solvent A) for 10 min prior to each new injection.

The Accucore C30 column (150 × 2 mm I.D., particle size: 2.6 µm, pore diameter: 150 Å) was obtained from ThermoFisher Scientific (ON, Canada). The mobile phase system consisted of solvent A (acetonitrile: H_2_O 60:40 v/v) and solvent B (isopropanol:acetonitrile:water 90:10:1 v/v/v) both containing 10 mM ammonium formate and 0.1% formic acid. C30-RPLC separation was carried out at 30 °C (column oven temperature) with a flow rate of 0.2 mL/min, and 10 µL of the lipid extraction suspended in chloroform:methanol (1:1 v/v) was injected onto the column. The following system gradient was used for separating the lipid classes and molecular species: 30% solvent B for 3 min; then solvent B increased to 43% over 5 min, then to 50% B in 1 min, then to 90% B over 9 min, then to 99% B over 8 min and finally kept at 99% B for 4 min. The column was re-equilibrated to starting conditions (70% solvent A) for 5 min prior to each new injection.

The method was applied in parallel as complementary system to take advantages of both (i) HILIC column to screen any novel lipid class present in the samples and (ii) C30-RPLC to confirm the HILIC results and provide further information on the modified lipids.

### High resolution tandem mass spectrometry analysis

Lipid analyses were carried out using a Q-Exactive Orbitrap mass spectrometer controlled by X-Calibur software 4.0 (ThermoScientific, MO, USA) with an automated Dionex UltiMate 3000 UHPLC system controlled by Chromeleon software. The following parameters were used for the Q-Exactive mass spectrometer - sheath gas: 40, auxiliary gas: 2, ion spray voltage: 3.2 kV, capillary temperature: 300 °C; S-lens RF: 30 V; mass range: 200–2000 m/z; full scan mode at a resolution of 70,000 m/z; top-20 data dependent MS/MS at a resolution of 35,000 m/z and collision energy of 35 (arbitrary unit); isolation window: 1 *m/z*; automatic gain control target: 1e5. The instrument was externally calibrated to 1 ppm using ESI negative and positive calibration solutions (ThermoScientific, MO, USA). Tune parameters were optimized using a mixture of lipid standards (Avanti Polar Lipids, Alabama, USA) in both negative and positive ion modes.

## Results and Discussion

### Analysis of the complex lipid standard mixture using HILIC or C30-RPLC-HRMS/MS

**Figure 1:**
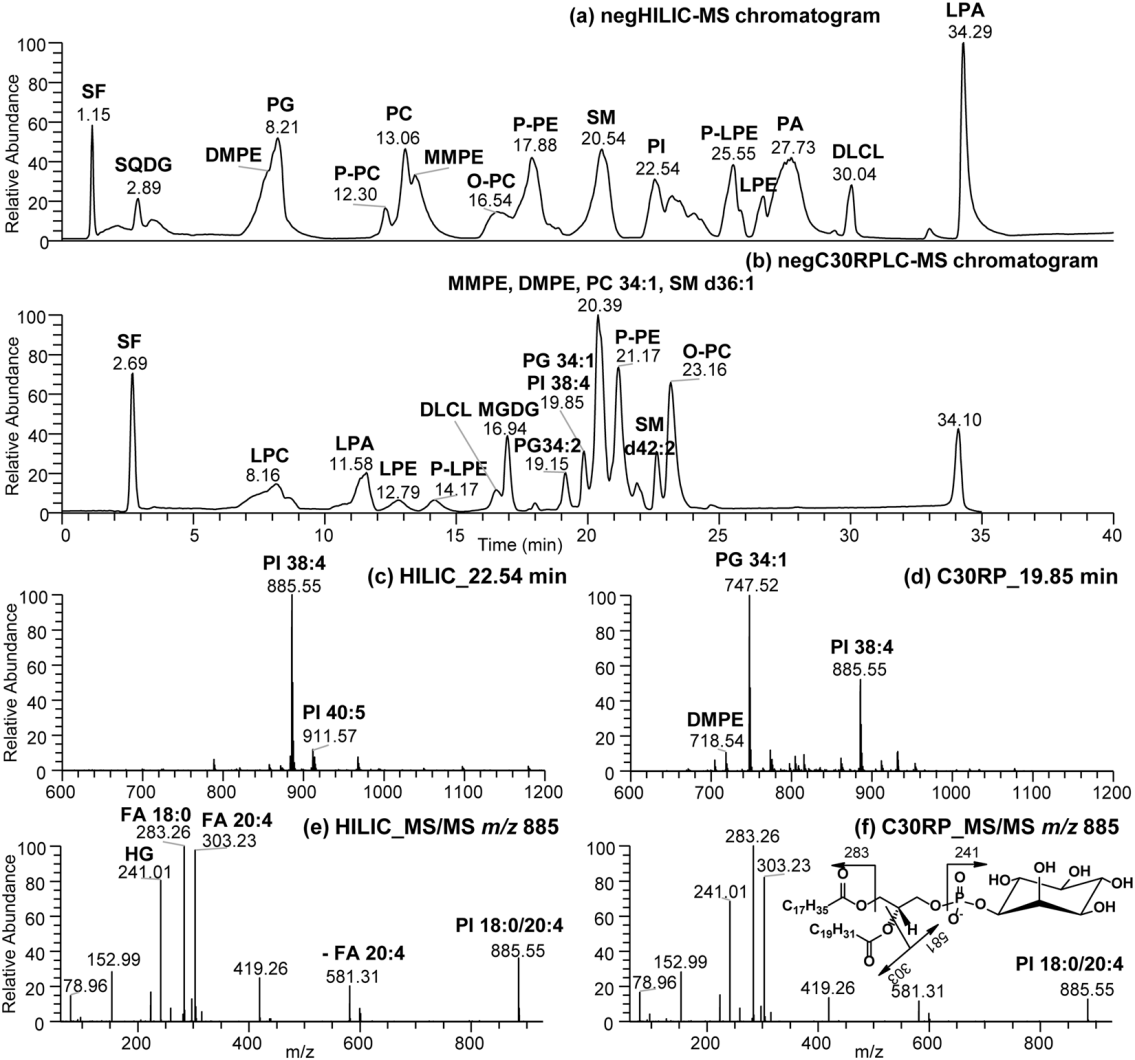
UHPLC-MS chromatogram in negative ion mode of the standard lipid mixture obtained after (**a**) HILIC and (**b**) C30-RPLC. (**c**) HILIC-MS spectrum of PI eluted at 22.54 min, (**d**) C30-RPLC-MS spectrum showing PI, PG and DMPE eluted at 19.85 min. (**e**,**f**) MS/MS spectra of PI 18:0/20:4 from HILIC and C30RPLC, respectively.

We utilized a solvent system consisting of acetonitrile, water and ammonium acetate buffer for HILIC chromatography, which efficiently separated 15 classes of the lipids present in the complex lipid standard mix in negative ion mode (Fig. 1a). The resolution of the lipid classes (inter-class differentiation) based on their headgroup composition occurred in the following order: SQDG, PG, DMPE, P-PC, PC, MMPE, O-PC, P-PE, SM, PI, P-LPE, LPE, PA, DLCL, and finally LPA (Fig. 1a). In comparison to HILIC, a C30-RPLC coupled with HESI-MS in negative ion mode is shown in Fig. 1b for the same complex lipid standards mixture. It is noted here that the buffer conditions were different between the two platforms. Formate buffer was used in the C30RP method whereas acetate buffer was utilized in the HILIC method for column optimization. Therefore, some lipid classes form acetate adducts [M + CH_3_COO]^−^ under HILIC-MS conditions, and formate adducts [M + HCOO]^−^ when C30-RPLC-MS was performed. The observed mass (*m/z*) and ion types of each lipid standard from both chromatograms are included in Table S-1.

Examples of the HILIC-HESI-MS and C30-RPLC-MS spectra are shown in Fig. 1c,d. We observed PI 38:4 eluted at 22.54 min under HILIC conditions together with other PI species (Fig. 1c), whereas PI 38:4 was seen together with PG 34:1 and DMPE 32:0 under C30RP conditions (Fig. 1d). A typical MS2 fragmentation of PI 38:4 [M-H]^−^ *m/z* 885.55 ion was displayed in Fig. 1e,f with the characteristic ions for diagnosis of the inositol headgroup at *m/z* 241.01 and the two fatty acid composition *m/z* 283.26 (C18:0) and *m/z* 303.23 (C20:4). The fragmentation of PI was known to be more complicated and different from other phospholipid classes in which the intensity of *sn*-2 FA generated from PI is either relatively lower than or nearly equal to that of the *sn-*1 fatty acid ion^32,37^. The *m/z* 419.26 product ion arising from further inositol loss after initial C20:4 fatty acid loss (i.e. *m/z* 581.31) was occurring preferentially at the *sn*-2 position^37,38^. This preference was used to assign the positions of FA chains on the glycerol backbone of PI. Thus the regiochemical assignment was consistent with PI 18:0/20:4 from bovine liver included in the lipid standard mix. The HRMS/MS spectrum from HILIC (Fig. 1e) was compared with that from C30RP in (Fig. 1f), and all the major fragment ions were present in the two MS^2^ spectra as expected. HRMS/MS permitted the accurate assignment of the PI molecular species following both C30RP and HILIC chromatography.

**Figure 2:**
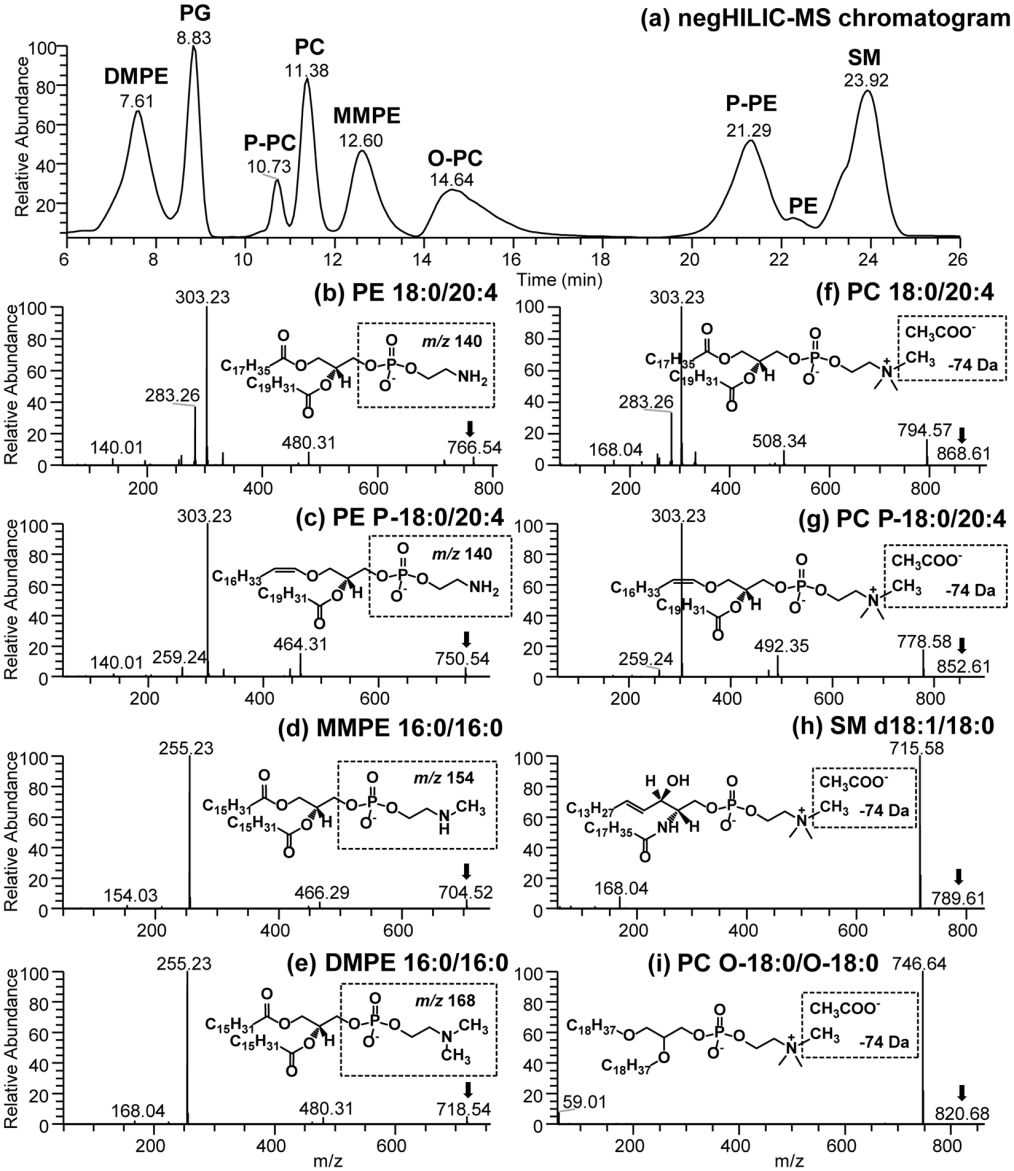
(**a**) HILIC-MS chromatogram of modified lipids showing separation and comparisons of PE, P-PE, MMPE, DMPE, PG, PC, O-PC, P-PC and SM lipid classes in negative ion mode. (**b**–**i**) HILIC-MS/MS spectra obtained from the complex lipid standard mix: (**b**) PE 18:0/20:4 [M-H]^−^ at *m/z* 766.54, (**c**) plasmalogen PE P-18:0/20:4 [M-H]^−^ *m/z* at 750.54, (**d**) MMPE 16:0/16:0 [M-H]^−^ at *m/z* 704.52, (**e**) DMPE 16:0/16:0 [M-H]^−^ at *m/z* 718.54, (**f**) PC 18:0/20:4 [M + CH_3_COO]^−^ at *m/z* 818.59, (**g**) plasmalogen PC P-18:0/20:4 [M + CH_3_COO]^−^ at *m/z* 852.61, (**h**) SM d18:1/18:0 [M + CH_3_COO]^−^ at *m/z* 789.61, and (**i**) diether PC O-18:0/O-18:0 [M + CH_3_COO]^−^ at *m/z* 820.68.

We assessed the efficiency of HILIC chromatography in resolving representative modified polar lipids in a complex standard mixture. Optimization of the mobile phase gradient using an isocratic elution period consisting of 5% solvent B from 5 min to 15 min improved the modified lipid subclasses separation in the first half of HILIC chromatogram (Fig. 2a). A clear separation of PE (22.25 min), P-PE (21.29), MMPE (12.60 min), DMPE (7.61 min), P-PC (10.73 min), O-PC (14.64), PC (11.38 min) and SM (23.92 min) standards was achieved. The modification of headgroup lowered the polarity of lipid compounds thereby reducing the retention times of MMPE and DMPE remarkably. High resolution mass spectrometry (HRMS) allowed for the identification of ether or ester linked lipid isobars, while the representative subclasses and fatty acid composition of the lipids present in the standard mixture were verified by HRMS/MS spectra as shown in Fig. 2(b–i). For example, *m/z* 140 is well-known to be characteristic of the un-modified PE lipid class representing the phosphoethanolamine headgroup^39^, and the two fatty acids seen at *m/z* 283.26 (C18:0) and 303.23 (C20:4) were derived from PE 18:0/20:4 [M-H]^−^ present in the complex standard mix (Fig. 2b). The position of FA 18:0 on *sn*-1 of the glycerol backbone was identified by a lyso-form fragment ion, *i*.*e*., LPE 18:0 seen at *m/z* 480.31 in Fig. 2b (listed as characteristic ion for PE in Table S-1. Conversely, methylated PE headgroups are characterized by an increase of 14 Da for *N-*monomethyl phosphoethanolamine ion (*m/z* 154 for MMPE in Fig. 2d) and 28 Da for *N*,*N*-dimethyl phosphoethanolamine ion (*m/z* 168 for DMPE headgroup in Fig. 2e). The product ion at *m/z* 168 is also present in PC and SM mass spectra associated with de-methylation of the phosphocholine headgroup and can be differentiated from PE by a companion of CH_3_COOCH_3_ loss (*i*.*e*., -74 Da observed in Fig. 2f–i).

Plasmalogen lipids were separated from diacyl species under HILIC conditions, including P-PE (21.29 min) and P-PC (10.73 min) as per the corresponding MS/MS spectra shown in Fig. 2c,g. The presence of the ether bonds also lowered the polarity of lipids and reducing their retention times under HILIC condition. As such, plasmalogens PC and PE eluted before their diacyl partners consistent with the confirmed identification by HRMS/MS spectra shown in Fig. 2b,c and Fig. 2f,g for comparison. Tandem mass spectrometry characterized both the plasmalogen and diacyl PC 18:0/20:4 headgroup by a neutral loss of 74 Da (*i*.*e*., -CH_3_COOCH_3_). This neutral loss forming the [M-CH_3_]^−^ product ion is known as a diagnostic ion for lipids with choline functional group in negative ion mode^31^. The transition from an ester to an ether bond resulted in the absence of *m/z* 283 representing stearic acid (C18:0) from PC P-18:0/20:4. The lyso-fragment ions from the *sn*-1 position seen at *m/z* 508.34 and *m/z* 492.35 represents the LPC 18:0 and LPC P-18:0 [M-CH_3_]^−^ ions, respectively (Fig. 2f,g). Sharing the same phosphocholine headgroup, SM species also produced characteristic loss of 74 Da with a companion of *m/z* 168, although they were readily distinguished from PC based on accurate mass of precursor ion and fragmentation (Fig. 2h). The diether PC O-18:0/O-18:0 standard produced very poor fragmentation due to the presence of double ether linkage, which only formed one major product ion at *m/z* 746.64 from the loss of CH_3_COOCH_3_ arisen from de-methylation of phosphocholine headgroup (*i*.*e*., −74 Da in Fig. 2i).

### Application of HILIC chromatography to analyze modified (methylated) PE lipids in the membrane of soil microbes

**Figure 3:**
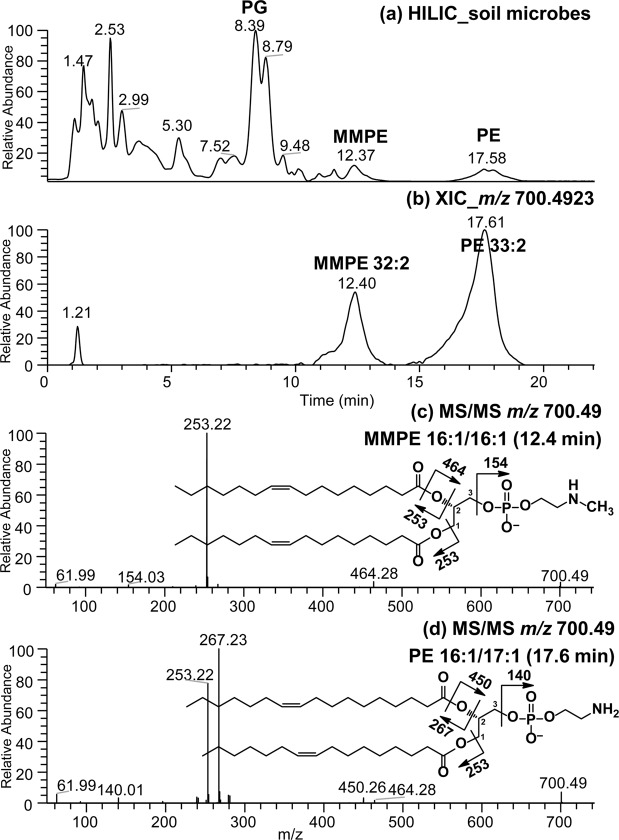
(**a**) HILIC-MS chromatogram of complex lipids extracted from soil microbes in negative ion mode. (**b)** Extracted ion chromatogram (XIC) of *m/z* 700.49, and (**c**–**d**) HILIC-HRMS/MS spectra of *m/z* 700.49 eluted at 12.40 min and 17.61 min representing MMPE 16:1/16:1 and PE 16:1/17:1 molecular species, respectively.

The complex lipid profile of soil microbes was examined using the HILIC method in negative ion mode (Fig. 3a). We observed the presence of monomethylated PE (MMPE) occurred at significant levels along with isomeric forms of diacyl PE within the cell membrane of soil microbes living under cool climatic conditions in podzolic soils (Fig. 3). The HILIC chromatography is efficient in separating the two PE subclasses, which allowed for facile identification and relative quantitation. LC-MS/MS spectra were used to assign the molecular species composition of both the monomethylated and diacyl PE subclasses (Table 1).

**Table 1:** Relative quantitation of MMPE and PE molecular species present in soil microbes resolved from HILIC-MS chromatograms in negative ion mode.

RT (min)	*m/z*	Prec. Ion	Molecular species	Diacyl species	% nmol
18.16	646.4820	[M-H]^−^	PE O-30:1	O-15:0/15:1	2.57 ± 0.07
17.68	660.4613	[M-H]^−^	PE 30:1	15:0/15:1	5.18 ± 0.14
18.32	662.4770	[M-H]^−^	PE 30-0	15:0/15:0	4.01 ± 0.09
17.95	672.4977	[M-H]^−^	PE P-32:1	P-16:0/16:1	2.48 ± 0.04
12.51	674.4767	[M-H]^−^	MMPE 30:1	15:1-15:0	0.66 ± 0.02
17.87	674.4767	[M-H]^−^	PE 31:1	16:1-15:0	6.80 ± 0.04
12.36	686.4767	[M-H]^−^	MMPE 31:2	15:1-16:1	0.18 ± 0.01
17.61	686.4767	[M-H]^−^	PE 32:2	16:1/16:1	11.33 ± 0.07
12.67	688.4925	[M-H]^−^	MMPE 31:1	16:1/15:0	2.26 ± 0.03
18.01	688.4925	[M-H]^−^	PE 32:1	16:0-16:1, 17:1/15:0	12.08 ± 0.06
12.40	700.4925	[M-H]^−^	MMPE 32:2	16:1/16:1	3.61 ± 0.17
17.60	700.4925	[M-H]^−^	PE 33:2	16:1-17:1	5.08 ± 0.15
12.76	702.5080	[M-H]^−^	MMPE 32:1	16:0/16:1, 17:1/15:0, 18:1/14:0	2.77 ± 0.10
18.22	702.5080	[M-H]^−^	PE 33:1	16:0/17:1, 17:0-16:1, 18:1/15:0	8.53 ± 0.06
12.41	714.5080	[M-H]^−^	MMPE 33:2	17:1-16:1	1.54 ± 0.08
17.79	714.5080	[M-H]^−^	PE 34:2	18:1-16:1, 17:1/17:1, 16:0/18:2	9.65 ± 0.28
12.87	716.5239	[M-H]^−^	MMPE 33:1	16:0-17:1, 17:0-16:1, 18:1/15:0	2.40 ± 0.06
18.53	716.5239	[M-H]^−^	PE 34:1	17:1/17:0, 16:0/18:1, 18:0/16:1	3.75 ± 0.10
12.50	728.5239	[M-H]^−^	MMPE 34:2	18:1/16:1, 17:1/17:1	2.59 ± 0.07
18.01	728.5239	[M-H]^−^	PE 35:2	18:1/17:1, 16:1-19:1	2.47 ± 0.02
17.52	740.5239	[M-H]^−^	PE 36:3	18:1-18:2	0.85 ± 0.07
12.63	742.5395	[M-H]^−^	MMPE 35:2	17:1-18:1	0.76 ± 0.03
18.34	742.5395	[M-H]^−^	PE 36:2	18:1/18:1	5.18 ± 0.19
12.99	756.5551	[M-H]^−^	MMPE 36:2	18:1/18:1	1.94 ± 0.01
19.15	756.5551	[M-H]^−^	PE 37:2	18:1-19:1	1.32 ± 0.01
				**Total% MMPE**	**18.73 ± 0.50**
				**Total% PE**	**81.27 ± 0.50**
				**Total%**	**100**

Values (percent by relative peak area) represent means ± standard errors, n = 4 per experimental replicate. PE represents phosphatidylethanolamine, MMPE represents N-monomethyl phosphatidylethanolamine subclass. The lipid components in the table are arranged based on *m/z* of the molecular species. If the position of fatty acyl chains on glycerol backbone was assigned, the molecular species was denoted with a slash *sn*-1/*sn*-2, if not a hyphen was used (e.g. PE 16:1-17:1) where the order of FA implies the more dominant molecular species and the hyphen indicates the presence of both *sn*-isomers^8^. Analysis was conducted using HILIC-HRMS/MS in negative ion mode.

For example, two isomers at *m/z* 700.49 were clearly resolve following HILIC chromatography (Fig. 3b). The assignments were made as follows: *m/z* 140.01 and 154.03 fragment ions were used to distinguish between PE and MMPE head groups. It is known for PE lipids that the pathways leading to the formation of the carboxylate anion and of the ions corresponding to a ketene loss are sterically more favorable at the *sn-*2 position under low-energy CID^37,40^. The modified monomethyl PE lipid in soil microbes was identified as MMPE 16:1/16:1 which produced only one carboxylate anion *m/z* 253.22 from C16:1 fatty acid (Fig. 3c). The *sn*-2 ketene loss of C16:1 forming *m/z* 464.28 ion (Fig. 3c) same as the minor ion observed in Fig. 3d.

The carboxylate anions were seen at *m/z* 253.22 (C16:1) and *m/z* 267.23 (C17:1) from PE 33:2 to give the molecular species composition of PE 16:1–17:1 (Fig. 3d). Higher abundance of *m/z* C17:1 carboxylate anion together with major lyso-form fragment ion observed at *m/z* 450.26 (C17:1 *sn*-2 ketene loss as depicted in Fig. 3d) indicated PE 16:1/17:1 as the dominant isomer. Also a minor ion observed at *m/z* 464.28 in Fig. 3d representing the presence of PE 17:1/16:1 molecular species in the sample (Table 2).

**Table 2:** Relative composition of major (acyl)phosphatidylglycerol molecular species present in silage corn roots following cultivation in cool climatic conditions.

*m/z*	Lipid Ion	Molecular species	Di/triacyl species	% nmol
765.52	[M + Na-2H]^−^	PG 34:3	16:0/18:3	6.22 ± 0.20
767.53	[M + Na-2H]^−^	PG 34:2	16:0/18:2	53.43 ± 0.84
789.52	[M + Na-2H]^−^	PG 36:5	18:2/18:3	2.36 ± 0.25
791.53	[M + Na-2H]^−^	PG 36:4	18:2/18:2	9.29 ± 0.26
795.56	[M + Na-2H]^−^	PG 36:2	18:2/18:0	3.49 ± 0.08
1003.75	[M + Na-2H]^−^	acylPG 50:3	16:0/18:3-(16:0)	1.19 ± 0.07
1005.76	[M + Na-2H]^−^	acylPG 50:2	16:0/18:2-(16:0)	17.99 ± 0.50
1029.76	[M + Na-2H]^−^	acylPG 52:4	18:2/18:2-(16:0), 16:0/18:2-(18:2)	4.12 ± 0.28
1031.77	[M + Na-2H]^−^	acylPG 52:3	18:1-18:2-(16:0),16:0-18:2-(18:1),	1.91 ± 0.14
			16:0-18:1-(18:2)	
			**Total% acylPG**	**25.22 ± 0.80**
			**Total% PG**	**74.78 ± 0.80**
			**Total%**	**100**

Values (percent by relative peak area) represent means ± standard errors, n = 4 per experimental replicate. PG represents phosphatidylglycerol, acylPG represents acylphosphatidylglycerol subclass. The lipid components in the table are arranged based on *m/z* of the molecular species [M + Na-2H]^−^ ions with the number before the colon representing total number of carbons, the numbers after the colon representing the total number of double bonds of all the fatty acyl chains composition (*e*.*g*., acylPG 50:2 represents acylPG molecules with 50 carbons and 2 double bonds on three acyl chains). The position of fatty acyl chains on glycerol backbone was denote with the order *sn*-1/*sn*-2-(*sn*-3′), hyphen was used if *sn*-1 and *sn*-2 were not assigned. Analysis was conducted using HILIC-HRMS/MS in negative ion mode.

Assignments for the major diacyl and methylated PE isomers found in soil microbes are listed in Table 1 along with their retention times. Extracted area counts were used for relative quantitation (Table 1). As such, MMPE accounted for 18.73% of the total PE lipid class composition observed in soil microbes living under cool climatic conditions in podzolic soils. These results demonstrate potential applications of the enhanced HILIC method for the inclusion of methylated PE (modified PE) during routine lipidomics analysis of complex biological samples.

### Application of the HILIC and C30-RPLC to detect acylPG in silage corn root samples

**Figure 4:**
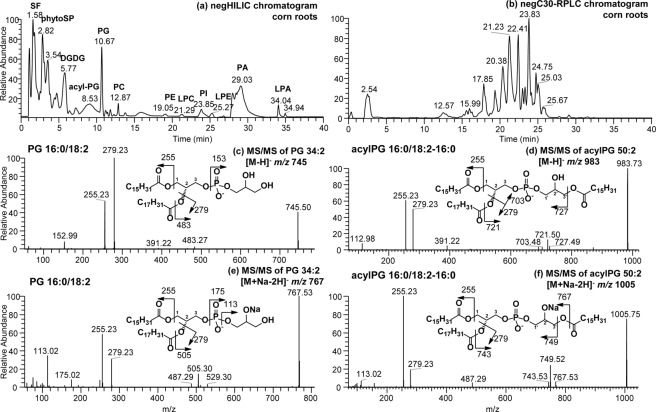
(**a**) HILIC and (**b**) C30RPLC-MS chromatograms of complex lipids observed in silage corn roots in negative ion mode. MS/MS spectra of PG 34:2 identified as (**c**) [M-H]^−^ ion at *m/z* 745.50 or (**e**) [M + Na-2H]^−^ ion at *m/z* 767.53 and acylPG 50:2 identified as (**d**) [M-H]^−^ ion at *m/z* 983.73 or (**f**) [M + Na-2H]^−^ ion at *m/z* 1005.75. Example of the structure and fragmentation of PG and acylPG are inserted in each MS/MS spectrum.

Membrane lipid composition in roots is of interest to study plant stress responses under various climatic conditions and crop management regimes^41^. HILIC chromatography was applied to assess silage corn root membrane lipid class composition when cultivated under cool climatic conditions (Fig. 4a). Three categories of lipids including phytosphingolipids (phytoSP), glycolipids and phospholipids (8 classes) were detected in the complex lipid mixture obtained from silage corn roots. The corn root membrane polar lipid classes were observed to consist of PG, PC, PE, LPC, PI, LPE, PA and LPA in which intra-class separation of individual molecular species can be partly resolved following HILIC-HRMS/MS analysis (see Fig. S1).

**Figure 5:**
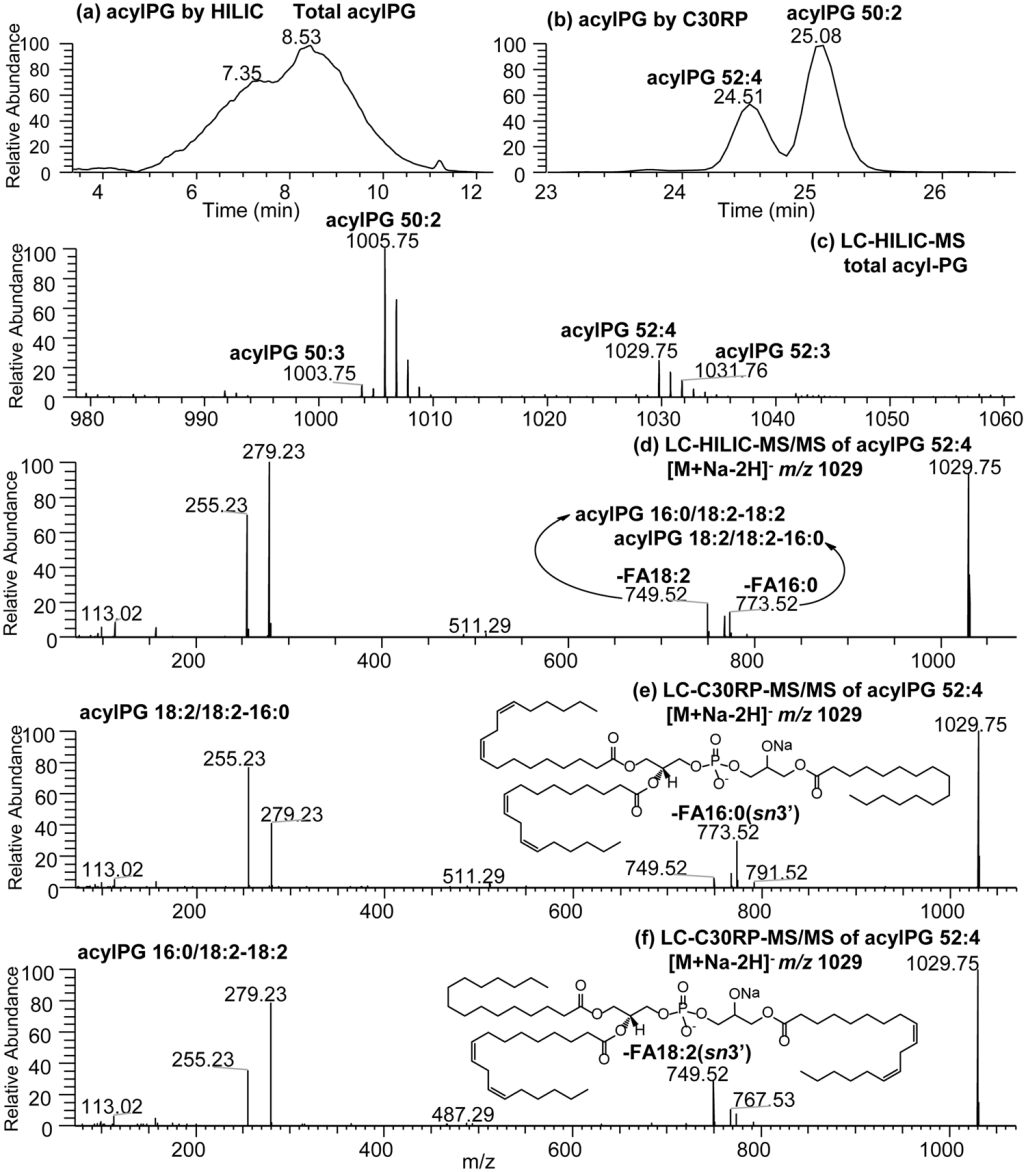
Extracted ion chromatogram (XIC) of acylPG in corn roots obtained from (**a**) HILIC and (**b**) C30-RPLC, (**c**) HILIC-MS of total acylPG. Corresponding HRMS/MS spectrum of *m/z* 1029.76 obtained from (**d**) HILIC and (**e**,**f**) C30-RPLC. Structures of acylPG are inserted in the MS/MS spectra.

Using this approach, we observe the presence of modified PG in corn roots. PG lipids are known to have important correlation with the plant sensitivity toward chilling temperature^13^. The modified PG was identified as acylphosphatidylglycerol (acylPG) following HRMS/MS analysis (Fig. 4). Both the modified (acylPG) and the un-modified PG were observed in the silage corn roots as sodiated [M + Na-2H]^−^ and deprotonated ([M-H]^−^) adducts (Table 2, Figs 4 and 5). It is important to note that no sodium buffer was added to the solvent system. Roots are known to have high levels of sodium from soil uptake, and appears to be the source of endogenous sodium that formed the adducts with PG^42^. The acylated modification occurred at the glycerol headgroup of PG, and were detected in pairs with the un-acylated form of PG. The modified headgroup resulted in acylPG being less polar than PG, and as such eluted before PG during HILIC chromatography. Thus, acylPG was observed to elute at 8.53 min immediately before the PG peak eluted at 10.67 min during HILIC chromatography (Fig. 4a). Confirmation of the identity of acylPG was done based on HRMS/MS fragmentation patterns (Fig. 4e,f). The PG head group was identified by its deprotonated [M-H]^−^ precursor ions (Fig. 4c,d) or sodiated adduct ions (Fig. 4e,f). The determination of fatty acyl chain positions on the glycerol backbone was based on relative abundance of the carboxylate anions and ketene losses arising from *sn*-1, *sn*-2 and *sn*-3′ positions (*sn*-3′ represents the acylated position at the phosphoglycerol headgroup). The mass spectral interpretation is in agreement with fragmentation of acylPG reported previously^12^. Of the total silage corn root PG and acylPG mass spectral intensity; 25.22% was from acylPG (Table 2).

The MS/MS spectrum of acylPG 50:2 at *m/z* 983.73 ([M-H]^−^) in Fig. 4d shows the most prominent loss of C18:2-ketene at *m/z* 721.50 which is diagnostic for the fatty acyl chain at the *sn*-2 position of the glycerol backbone. Additionally, *m/z* 255 and *m/z* 279 ions are diagnostic for C16:0 and C18:2 fatty acyl chains indicating only two types of fatty acids are present in this acylPG molecular species. Since C18:2 fatty acid was already identified at the *sn*-2 position of the glycerol moiety, it leaves both C16:0 fatty acyl chains at the *sn-*1 and *sn-*3′. This is consistent with Hsu *et al*. study^12^ which reported that the relative abundance of fatty acid loss at *sn*-1 or *sn*-3′ is higher than that for the ketene loss (i.e. *m/z* 727.49 in Fig. 4d corresponding to C16:0 acid loss). Intriguingly, the same compound as sodiated adduct [M + Na-2H]^−^ produced a more noticeable fragment ion (*m/z* 749.52 in Fig. 4f) for the loss of C16:0 fatty acid than for example the ketene loss and was assigned as the *sn-*3′ fatty acid. Thus, acylPG 50:2 in corn root membrane was assigned as acylPG 16:0/18:2-(16:0) in both [M-H]^−^ and [M + Na-2H]^−^ adducts. This also change the order of carboxylate anions which was reported as *sn-*2 > *sn-*3′ ≫ *sn-*1 from [M-H]^−^ to much higher relative abundance of *sn-*3′ ≫  *sn-*2 > *sn-*1 fatty acyl anions (*e*.*g*., *m/z* 255 and *m/z* 279 in Fig. 4e,f). As discussed earlier, the position of the fatty acyl chains in PGs from corn roots may have significance in terms of indicating their biosynthesis pathway and thus identifying their positional isomers are targeted using chromatography.

Analysis of acylPG using both HILIC and C30RP approaches in Fig. 5 demonstrated the usefulness of using C30RP complementary to HILIC to resolve the acylPG isomers. HILIC resulted in all the acylPG molecular species eluting as one peak and were resolved into individual molecular species following separation using high resolution tandem mass spectrometry (Fig. 5a,c). On the other hand, the molecular species were separated based on chain length and unsaturation using C30-RPLC (Fig. 5b). For example, acylPG 52:4 in corn root was observed from HILIC-HRMS/MS in Fig. 5d to exist as two isomers of acylPG 16:0/18:2-(18:2) and 18:2/18:2-(16:0). The presence of both isomers was assigned from *sn-*3′ C16:0 and C18:2 fatty acid losses, represented by almost the same abundance of *m/z* 773.52 and 749.52, respectively (Fig. 5d). Both acylPG 52:4 isomers were also observed following C30-RPLC to be present at the beginning and end of the peak eluting at 24.51 min (Fig. 5b) and their corresponding C30RPLC-MS/MS spectra further confirming the results obtained using HILIC. Major differences between fragmentation of the two positional acylPG isomers are seen in Fig. 5(e,f), including the opposite relative abundance of *m/z* 255.23 (C16:0) and 279.23 (C18:2) along with the characteristic loss of *sn-*3′ fatty acid, *i*.*e*., *m/z* 773.52 from acylPG 18:2/18:2-(16:0) and *m/z* 749.52 from acylPG 16:0/18:2-(18:2). For the latter, C18:2 was assigned at *sn-*2 position due to the relative abundance of *m/z* 255 and 279 and it is consistent with assignment from [M-H]^-^ fragmentation (see Figs S2 and S3).

This result demonstrates the application of the combined LC platforms to permit the detection of a new lipid in silage corn root during routine lipidomics. We also observed that using the HILIC complementary to C30 reverse phase chromatography aided in the identification of this headgroup modified PG in corn roots, while the C30RP was able to confirm the identity of the modified lipids with further intra-class separation of acylPG molecular species. Acylated phosphatidylglycerol (acylPG) are rare occurrence in plants, with a few exceptions reported in oats (*Avena sativa*)^43^ and *Arabidopsis* leaves^44^ as potential response to climatic conditions. The occurrence of acylPG observed in corn roots may well be associated with cultivation condition under cool climatic conditions in Newfoundland.

### Application of the HILIC chromatography to analyze regioisomers of lysophospholipids in kale leaf samples

**Figure 6:**
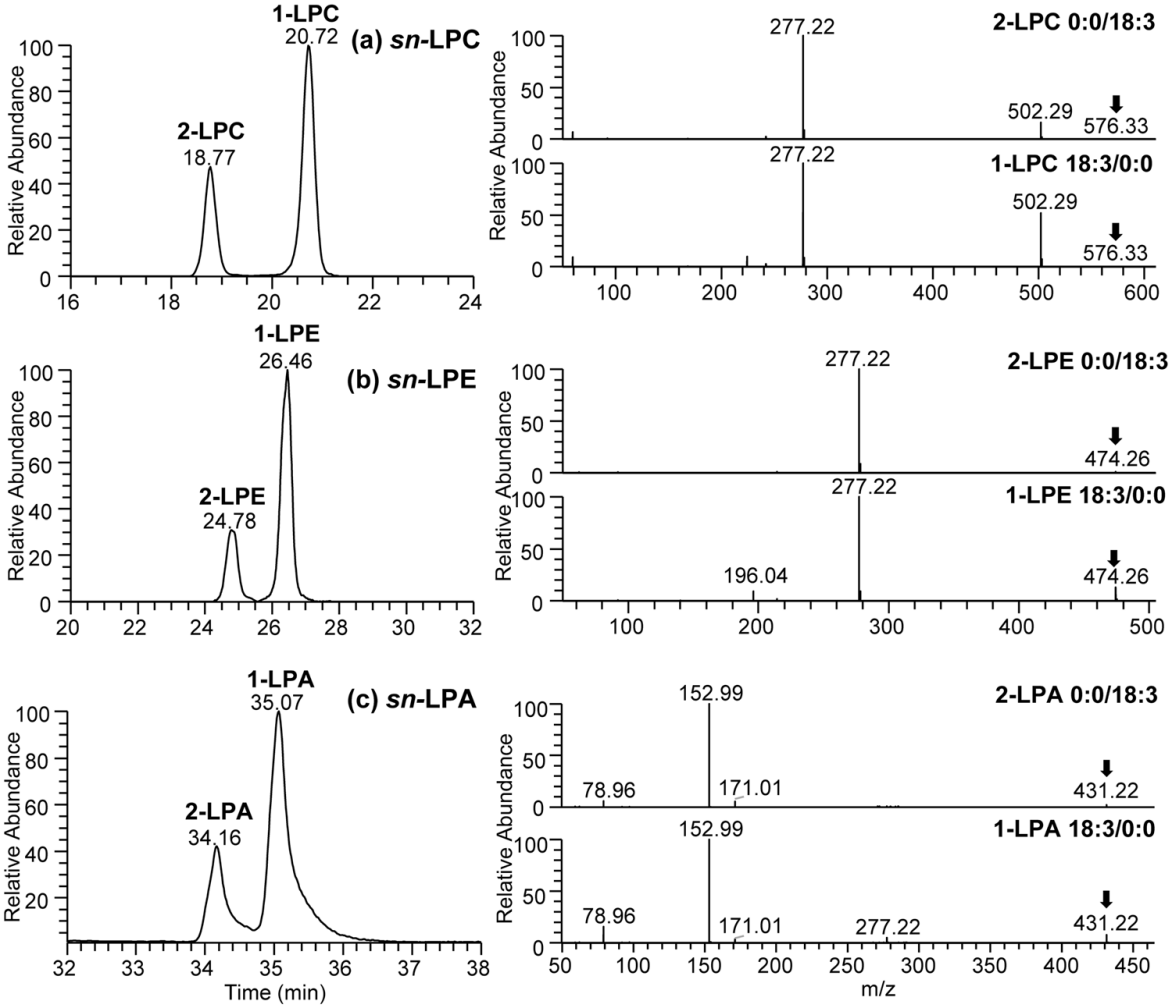
Extraction ion chromatography (XIC) and HRMS/MS spectra of (**a**) *sn-*LPC 18:3 regioisomers at *m/z* 576.33 (**b**) *sn-*LPE 18:3 regioisomers at *m/z* 474.26 and (**c**) *sn-*LPA 18:3 regioisomers at *m/z* 431.22 observed in kale leaves.

We also investigated HILIC chromatography to resolve regioisomers of lysophospholipids during routine analysis of kale lipidome following cultivation in different natural media amendments^35^. Following HILIC chromatography, not only the kale LPA, LPE and LPC lipids were clearly separated from each other, but also the regioisomers (differ based on the position of fatty acid at the *sn-*1 or *sn-*2 positions on the glycerol backbone) within each class of lysophospholipids were resolved. The *sn*-1 regioisomers were observed to have longer retention times, and higher relative abundance in comparison to *sn*-2 isomers consistent with previous findings^45^. HILIC-HRMS/MS spectra of the isomeric ion pairs are shown in Fig. 6, indicating a higher relative abundance of fatty acid ions formed by the cleavage of fatty acyl chain at the *sn*-2 position compared to the ions formed at the *sn*-1 position allows the differentiation of (lyso)phospholipid regioisomers.

The separation of the lysophospholipid classes and their regioisomers using HILIC chromatography permitted unambiguous identification and relative quantitation of each individual species present in the sample. Relative quantification of each regioisomer was based on the peak area obtained from the extracted ion chromatograms (XIC) along with the assigned retention times. The *sn*-1 regioisomer of lysophospholipids (1-LPE and 1-LPC) in kale leaves were observed at significantly higher level as compared to their *sn*-2 counterparts (Fig. 6 and Table S-2). Although the application of HILIC chromatography for separating regioisomers of lysophospholipids have been reported previously, this is the first time this information is reported for kale lysophospholipid regioisomers. The main purpose for this section is to demonstrate the applicability of the enhanced HILIC approach to facilitate the analysis of regioisomers present in lysophospholipids, as well as the analysis of modified phospholipids during routine lipidomics.

### C30-RPLC-HRMS/MS for the separation and identification of neutral lipids as a complementary platform to HILIC-HRMS/MS

**Figure 7:**
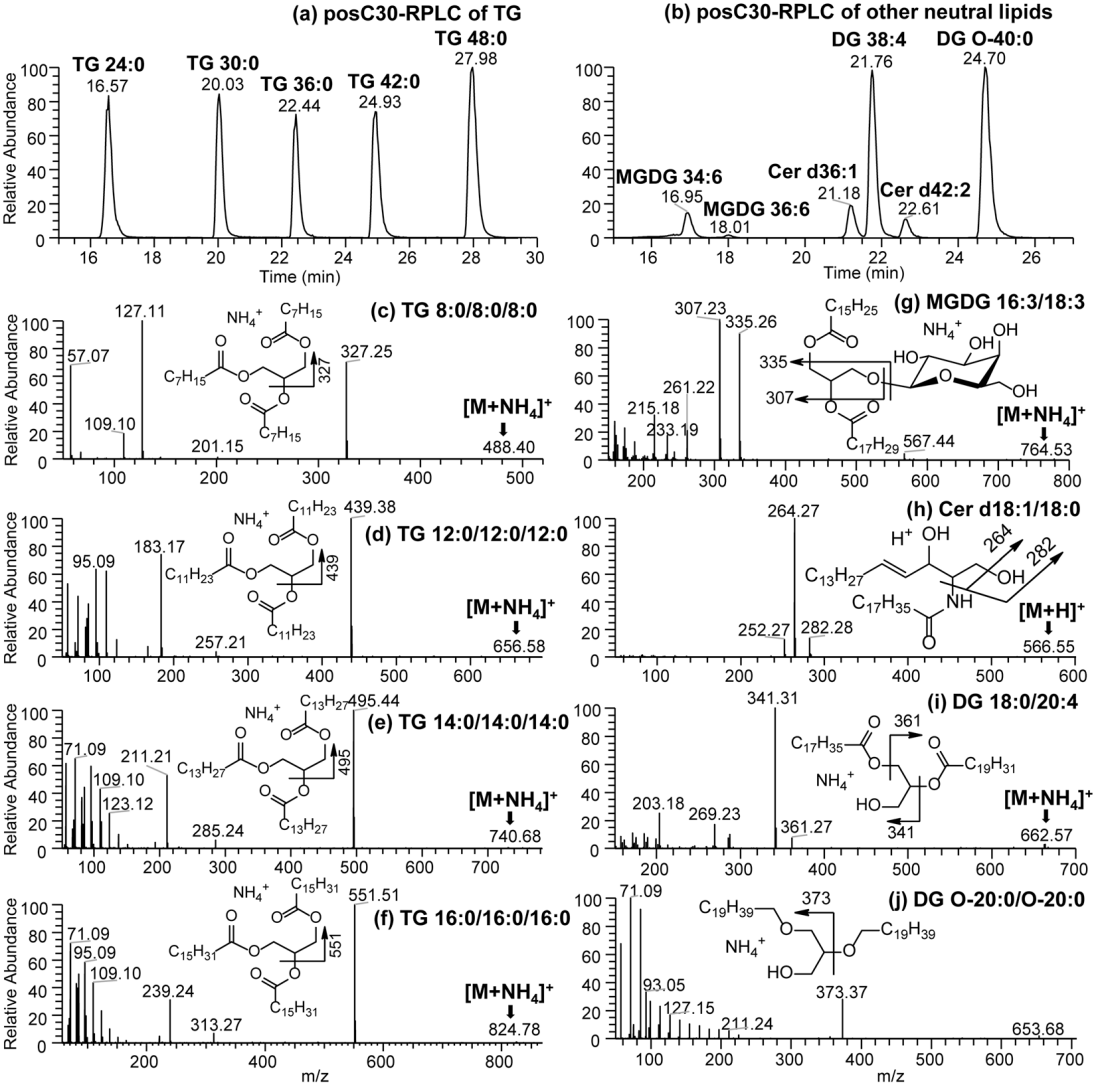
C30-RPLC-MS chromatogram showing separation of neutral lipid species in positive ion mode. (**a**) represents TG molecular species resolved based on chain length, (**b**) Resolution of DG, ceramides and glycolipid molecular species. C30-RPLC-MS/MS spectra of [M + NH_4_]^+^ ions representing lipid standards: (**c**) TG 8:0/8:0/8:0, (**d**) TG 12:0/12:0/12:0, (**e**) TG 14:0/14:0/14:0, (**f**) TG 16:0/16:0/16:0, (**g**) MGDG 16:3/18:3, (**h**) Cer d18:1/18:0 [M + H]^+^ ion, (**i**) DG 18:0/20:4 and (**j**) DG O-20:0/O-20:0.

One limitation of HILIC chromatography is the poor retention of neutral (non-polar) lipids such as mono/di/triacylglycerols, cholesterol, free fatty acids, etc. These neutral lipids tend to co-elute very early close to the solvent front (*i*.*e*., void volume peak) during HILIC. Thus C30-RPLC was chosen for analyzing neutral lipids as a complementary approach to HILIC. C30-RPLC has been compared with other reverse phase chromatographic methods (C8 and C18) and has been demonstrated to be very efficient in separating neutral lipids, especially triacylglycerols with high selectivity and geometric peak shapes^24,25,28^. In addition, the combination of C30RP with high resolution accurate mass spectrometry offer superior capabilities in separating neutral lipid isobars and isomers present in biological samples. We applied the C30-RPLC approach to separate the neutral lipids present in our standard mix using positive ion mode as shown in Fig. 7.

Triglyceride (TG) molecular species in the standard mixed were clearly resolved form each other based on the fatty acid composition and chain length ranging from TG 24:0 to TG 48:0 (Fig. 7a). The TG molecular species were observed as NH_4_^+^ adducts (Fig. 7e,f). For example, neutral loss of 161 Da in Fig. 7c is representative of C8:0 fatty acid loss from *m/z* 488.40 [TG 8:0/8:0/8:0 + NH_4_]^+^ ion, while *m/z* 551.51 ion in Fig. 7f corresponds to the neutral loss of FA 16:0 from TG 16:0/16:0/16:0. Fragment ions seen at *m/z* 239.24, *m/z* 313.27 and *m/z* 551.51 in Fig. 7f were assigned as ketene ions [FA 16:0 + H–H_2_O]^+^, [MG 16:0 + H–H_2_O]^+^ and [DG 32:0 + H–H_2_O]^+^, respectively. Using the typical product ions observed in positive ion mode for TG, complete FA profiles of each TG molecular species were elucidated consistent with the rules observed for TG fragmentation^46,47^. The diglycerides (DG) were also observed to be clearly resolved from each other following C30-RPLC-MS/MS regardless of linkages present in the structure. Both ether (O-DG) and ester linked DG molecular species were included in the lipid standard mix, and were observed as [O-DG + NH_4_]^+^ and [DG + NH_4_]^+^ at *m/z* 670.71 and *m/z* 662.57 respectively (Fig. 7i,j). The loss of water [H_2_O + NH_3_] (*i*.*e*., −35 Da) and the formation of MG fragment ions are characteristic for the identification of DG. For example, [MG 18:0 + H–H_2_O]^+^ at *m/z* 341.31 and [MG 20:4 + H–H_2_O]^+^ at *m/z* 361.27 were formed from DG 18:0/20:4 [M + NH_4_]^+^ present in the lipid standard mixture. The change of ester to ether linkage in O-DG resulted in the loss of alcohol (*m/z* 373.37) instead of a neutral fatty acid and is diagnostic of ether linked DG molecular species (Fig. 7j). The glycolipids were also observed to be clearly resolved from the TG, DG, and ceramide species following C30-RPLC-MS/MS analysis (Fig. 7a–j). MGDG molecular species were observed to elute between 16.95 and 18.01 minutes followed by Cer d36:1 at 21.18 minutes, DG 38:4 at 21.76 minutes, Cer d42:2 at 21.61 minutes and O-DG at 24.70 minutes (Fig. 7b). The TGs were interspersed between these molecular species and were clearly resolved before or after these species and classes based on the chain length (Fig. 7a,b).

**Figure 8:**
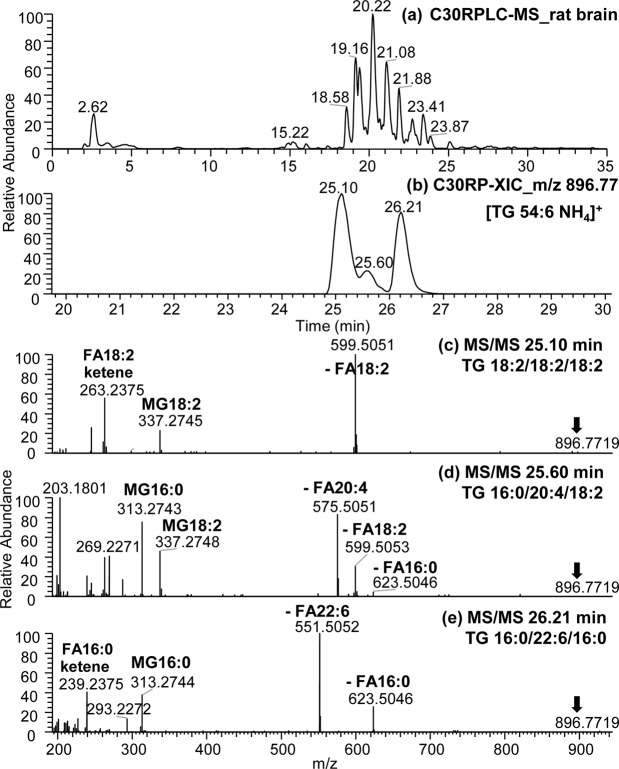
(**a**) C30-RPLC-HESI-MS chromatogram of rat brain lipids analyzed in positive ion mode. (**b**) Extracted ion chromatogram (XIC) showing separation of TG 54:6 isomers in rat brain, (**c**–**e**) C30-RPLC-HRMS/MS spectra of *m/z* 896.77 [M + NH_4_]^+^ precursor ion representing three isomers of (**c**) TG 18:2/18:2/18:2, (**d**) TG 16:0/20:4/18:2 and (**d**)TG 16:0/22:6/16:0 eluting at 25.10, 25.60 and 26.21 minutes, respectively.

Applying this approach to rat brain samples (Fig. 8), we demonstrated that C30-RPLC-MS/MS was efficient in resolving brain neutral lipids consistent with previous reports in the literature using this (C30) and other reverse phase (C8 or C18) columns^24,48^. Rat brain triglycerides are presented as an example of the C30-RPLC technique to analyze neutral lipids in biological samples. High resolution of rat brain TG isomers was accomplished using the C30-RP separation (Fig. 8b–e). For example, the extracted ion chromatogram shown in Fig. 8b of *m/z* 896.77 observed in rat brain TG gave three clearly resolved peaks eluted at 25.10, 25.60 and 26.21 mins (Fig. 8b) corresponding to three isomers (Fig. 8c–e) of [TG 54:6 + NH_4_]^+^. As elucidation from the neutral lipid standard mixture; the composition and position of fatty acyl chains in each TG molecular species can be identified by (*i*) the neutral loss of FA (as ammonium adducts), (*ii*) the formation of MG ion (in the form of [MG + H–H_2_O]^+^ product ions) and (*iii*) FA ketene ion (*i*.*e*., [FA + H–H_2_O]^+^)^46^. Using this as a guide, the three [TG 54:6 + NH_4_]^+^ isomers were assigned as TG 18:2/18:2/18:2, TG 16:0/20:4/18:2 and TG 16:0/22:6/16:0 molecular species (Fig. 8c–e). The purpose of this section is to demonstrate that using C30-RPLC-HRMS/MS as a complementary technique to HILIC-HRMS/MS is a suitable approach to analyze neutral lipids, modified lipids, as well as, regioisomers (*i*.*e*., *sn*-positional isomers) of lipids during routine lipidomics of a wide range of biological samples (microbes, plants and animals).

## Conclusion

In this work, we present the utilization of two enhanced high resolution liquid chromatography platforms coupled to high resolution accurate mass tandem mass spectrometry, for the analysis of modified lipids and regioisomers during routine lipidomics analysis. We also demonstrate that this platform is suitable for investigating the lipidome across different biological samples (animals, plants and microbes). HILIC separates lipids into classes including modified headgroups according to their polarity and electrostatic interactions. Regioisomers of *sn*-LPE, *sn*-LPC and *sn*-PI in kale leaves were separated and quantified using HILIC in the negative ion mode, while isomeric structures of neutral lipids, i.e., triacylglycerols in rat brain were demonstrated with C30-RPLC operated in the positive ion mode. In the current study, an enhanced HILIC method was demonstrated to be superior in resolving modified lipids present in a complex standard mixture. When we applied the enhanced approached during routine lipidomics to a range of biological samples we observed the following:

1. Discovery of modified PG (acylPG) in silage corn roots as potential response to the cultivation condition under cool climate.
2. The presence of high levels of monomethylated PE in the membrane of soil microbes living under cool climatic conditions in podzolic soils.
3. The composition and relative amounts of different regioisomers of lysophospholipids in kale leaves.

On the other hand, the optimized C30RP chromatography permitted excellent intra-class resolution (separation) of lipid isomers with different fatty acid composition or head group modification. The combination of both chromatography as complementary platform with high resolution tandem mass spectrometry allowed excellent resolution of lipid isomers or isobars, and therefore accurately distinguish di/triglycerides, plasmalogens and ether iso-forms of lipids in a diverse mix of biological samples. The work presented in this study demonstrated that one can use HILIC complementary with C30 reverse phase ultra-high performance liquid chromatography and high resolution accurate mass spectrometry as a high throughput lipidomic platform to analyze complex lipids (isobars, isomers, linkage or modified headgroup) in diverse biological systems. We believe this method could facilitate the inclusion and analysis of some complex lipid classes that are subject of more targeted lipid analysis into a more routine lipidomic analyses; and maybe of value to the general scientific community.

## Supplementary information

Supplementary Information
