# Supplementary material for: Targeting Modified Lipids during Routine Lipidomics Analysis using HILIC and C30 Reverse Phase Liquid Chromatography coupled to Mass Spectrometry
Source: Sci Rep. 2019 Mar 25;9:5048. doi: 10.1038/s41598-019-41556-9 (PMC6433904; doi:10.1038/s41598-019-41556-9)
Supplement: Supplementary file 1 — Supplementary Information [file 41598_2019_41556_MOESM1_ESM.docx]

**Supplementary Information**

*for*

Targeting Modified Lipids during Routine Lipidomics Analysis using HILIC and C30 Reverse Phase Liquid Chromatography coupled to Mass Spectrometry

Thu Huong Pham^1^, Muhammad Zaeem^1^, Tiffany A. Fillier, Muhammad Nadeem^1,3^, Natalia P. Vidal^1^,

Charles Manful^1^, Sukhinder Cheema^2^, Mumtaz Cheema^1^, Raymond H. Thomas^1^

^1^ School of Science and the Environment/ Boreal Ecosystem Research Initiative, Grenfell Campus, Memorial University of Newfoundland, Corner Brook, Newfoundland, A2H 5G4, Canada.

^2^ Department of Biochemistry, Memorial University of Newfoundland, St John's, Newfoundland, A1B 3X9, Canada.

^3^ Department of Environmental Sciences, COMSATS University of Islamabad, Vehari 61100, Pakistan

**Table S-1**. Lipid standards used in the optimization of the UHPLC-HILIC/C30RP-HESI-MS conditions. Information represents the molecular weight (*m/z*), ion type and LC-MS retention times of each lipid standard detected by mass spectrometry in negative ion mode under HILIC and C30 reverse phase conditions. Average retention time and standard deviation were calculated from four replicated lipid standard mixtures.

| **Lipid Class** | **Characteristic Ions** | **Lipid standards**  **Molecular species** | **HILIC chromatogram** | | | **C30RP chromatogram** | | |
| --- | --- | --- | --- | --- | --- | --- | --- | --- |
|  |  |  | ***m/z*** | **Ion type** | **RT (min)** | ***m/z*** | **Ion type** | **RT (min)** |
| **SQDG** | HG: *m/z* 225,  FA: *m/z* 255 | SQDG 16:0/16:0 | 793.51 | [M-H]^-^ | 2.90±0.05 | 793.51 | [M-H]^-^ | 19.37±0.01 |
| **PC** | HG: *m/z* 168,  M-CH_3_: *m/z* 794,  (*sn*-1) LPC: 508  FA: 283, 303 | PC 18:0/20:4 | 868.61 | [M+CH_3_COO]^-^ | 13.05±0.02 | 854.59 | [M+HCOO]^-^ | 20.27±0.01 |
| **P-PC** | HG: *m/z* 168,  M-CH_3_: *m/z* 778  (*sn*-1) P-LPC: *m/z* 492  (*sn*-2) FA: *m/z* 303 | PC P-18:0/20:4 | 852.61 | [M+CH_3_COO]^-^ | 12.27±0.03 | 838.59 | [M+HCOO]^-^ | 20.81±0.02 |
| **O-PC** | HG: *m/z* 168,  M-CH_3_: *m/z* 746 | PC O-18:0/O-18:0 | 820.68 | [M+CH_3_COO]^-^ | 16.62±0.09 | 806.66 | [M+HCOO]^-^ | 23.17±0.03 |
| **PE** | HG: *m/z* 140,  (*sn*-1) LPE: 480  FA: 283, 303 | PE 18:0/20:4 | 766.54 | [M-H]^-^ | 18.02±0.06 | 766.54 | [M-H]^-^ | 20.69±0.02 |
| **P-PE** | HG: *m/z* 140,  (*sn*-1) P-LPE: 464  (*sn*-2) FA: 303 | PE P-18:0/20:4 | 750.54 | [M-H]^-^ | 17.85±0.04 | 750.54 | [M-H]^-^ | 21.16±0.03 |
| **MMPE** | HG: *m/z* 154,  (*sn*-1) MMLPE: *m/z* 466,  FA: *m/z* 255 | MMPE 16:0/16:0 | 704.52 | [M-H]^-^ | 13.59±0.01 | 704.52 | [M-H]^-^ | 20.51±0.01 |
| **DMPE** | HG: *m/z* 168,  (*sn*-1) DMLPE: *m/z* 480,  FA: *m/z* 255 | DMPE 16:0/16:0 | 718.54 | [M-H]^-^ | 7.68±0.05 | 718.54 | [M-H]^-^ | 20.39±0.01 |
| **PG** | HG: *m/z* 153,  (*sn*-1) LPG: *m/z* 511,  FA: *m/z* 283, 303 | PG 18:0/20:4 | 797.53 | [M-H]^-^ | 8.20±0.04 | 797.53 | [M-H]^-^ | 19.85±0.01 |
| **PI** | HG: *m/z* 241, 153, 78  LPI(-H_2_O): *m/z* 581  FA: *m/z* 283, 303 | PI 18:0/20:4 | 885.55 | [M-H]^-^ | 22.54±0.06 | 885.55 | [M-H]^-^ | 19.85±0.01 |
| **SM** | HG: *m/z* 168,  M-CH_3_: *m/z* 715 | SM d18:1/18:0 | 789.61 | [M+CH_3_COO]^-^ | 20.54±0.06 | 775.60 | [M+HCOO]^-^ | 20.40±0.01 |
| **LPA** | HG: *m/z* 78, 153 | LPA 20:4 | 457.24 | [M-H]^-^ | 34.20±0.48 | 457.24 | [M-H]^-^ | 7.34±0.10 |
| **LPC** | HG: *m/z* 168,  M-CH_3_: *m/z* 506,  FA: *m/z* 281 | LPC 18:1 | 580.36 | [M+CH_3_COO]^-^ | 20.76±0.01 | 566.34 | [M+HCOO]^-^ | 8.63±0.01 |
| **LPE** | HG: *m/z* 140, 196  FA: *m/z* 283 | LPE 18:0 | 480.31 | [M-H]^-^ | 26.70±0.04 | 480.31 | [M-H]^-^ | 12.80±0.02 |
| **P-LPE** | HG: *m/z* 140, 78, 196,  Plasmenyl ion: *m/z* 267 | LPE P-18:0 | 464.31 | [M-H]^-^ | 25.46±0.02 | 464.31 | [M-H]^-^ | 14.18±0.04 |
| **DLCL** | HG: *m/z* 78, 153,  LPA: *m/z* 433  FA: *m/z* 279 | DLCL 18:2/18:2 | 923.51 | [M-H]^-^ | 30.01±0.03 | 923.51 | [M-H]^-^ | 16.53±0.05 |
| **CL** | HG: *m/z* 153,  PA: *m/z* 695,  LPA: *m/z* 433,  FA: *m/z* 279 | CL 18:2/18:2/18:2/18:2 | 1447.97 | [M-H]^-^ | 43.55±0.32 | 1447.97 | [M-H]^-^ | 24.00±0.08 |

RT = retention time, HESI= Heated electrospray ionization, HILIC = hydrophilic interaction chromatography, RP = reverse phase, HG = head-group, FA = fatty acid, SQDG = sulfoquinovosyldiacylglycerol, PE = phosphatidylethanolamine, P-PE = plasmalogen phosphatidylethanolamine, LPE = lysophosphatidylethanolamine, P-LPE = plasmalogen lysophosphatidylethanolamine, MMPE = N-monomethyl-phosphatidylethanolamine, DMPE = N,N-dimethyl-phosphatidylethanolamine, DMLPE = N,N-dimethyl-lysophosphatidylethanolamine, PG = phosphatidylglycerol, LPG = lysophosphatidylglycerol, PC = phosphatidylcholine, P-PC = plasmalogen phosphatidylcholine, O-PC = O-alkyl ether phosphatidylcholine, LPC = lysophosphatidylcholine, O-LPC = O-alkyl ether lysophosphatidylcholine, SM = sphingomyelin, PI = phosphatidylinositol, PA = phosphatidic acid, LPA = lysophosphatidic acid, DLCL = dilysocardiolipin, CL = cardiolipin.

**Table S-2.** Relative quantitation of *sn*-regioisomers of lysophospholipids (LPA, LPE, and LPC) present in kale leaves and resolved by HILIC-MS in negative ion mode.

| **RT (min)** | **Lipid class/Ion type** | ***m/z*** | **Molecular species** | **Diacyl species** | **% Intensity** |
| --- | --- | --- | --- | --- | --- |
| 34.24 | **LPA** | 431.22 | 1-LPA 18:3 | 18:3/0:0 | 27.58±0.66 |
|  | [M-H]^-^ | 431.22 | 2-LPA 18:3 | 0:0/18:3 | 12.20±0.44 |
|  |  | 433.24 | 1-LPA 18:2 | 18:2/0:0 | 33.43±0.35 |
|  |  | 433.24 | 2-LPA 18:2 | 0:0/18:2 | 13.53±0.43 |
|  |  | 435.25 | 1-LPA 18:1 | 18:1/0:0 | 9.83±0.37 |
|  |  | 435.25 | 2-LPA 18:1 | 0:0/18:1 | 3.44±0.19 |
|  |  |  |  | **Total% 1-LPA** | **70.84±0.30** |
|  |  |  |  | **Total% 2-LPA** | **29.16±0.30** |
|  |  |  |  | **Total%** | **100** |
| 26.46 | **LPE** | 452.28 | 1-LPE 16:0 | 16:0/0:0 | 40.01±0.30 |
|  | [M-H]^-^ | 474.26 | 1-LPE 18:3 | 18:3/0:0 | 13.71±0.30 |
|  |  | 474.26 | 2-LPE 18:3 | 0:0/18:3 | 4.22±0.15 |
|  |  | 476.28 | 1-LPE 18:2 | 18:2/0:0 | 21.16±0.09 |
|  |  | 476.28 | 2-LPE 18:2 | 0:0/18:2 | 5.86±0.12 |
|  |  | 478.29 | 1-LPE 18:1 | 18:1/0:0 | 7.93±0.12 |
|  |  | 478.29 | 2-LPE 18:1 | 0:0/18:1 | 0.65±0.01 |
|  |  | 480.31 | 1-LPE 18:0 | 18:0/0:0 | 6.47±0.16 |
|  |  |  |  | **Total% 1-LPE** | **89.27±0.30** |
|  |  |  |  | **Total% 2-LPE** | **10.73±0.30** |
|  |  |  |  | **Total%** | **100** |
| 20.76 | **LPC** | 554.35 | 1-LPC 16:0 | 16:0/0:0 | 27.21±0.60 |
|  | [M+CH_3_COO]^-^ | 576.33 | 1-LPC 18:3 | 18:3/0:0 | 19.29±0.51 |
|  |  | 576.33 | 2-LPC 18:3 | 0:0/18:3 | 8.08±0.33 |
|  |  | 578.35 | 1-LPC 18:2 | 18:2/0:0 | 20.10±0.05 |
|  |  | 578.35 | 2-LPC 18:2 | 0:0/18:2 | 7.92±0.18 |
|  |  | 580.36 | 1-LPC 18:1 | 18:1/0:0 | 8.71±0.17 |
|  |  | 580.36 | 2-LPC 18:1 | 0:0/18:1 | 3.54±0.05 |
|  |  | 582.38 | 1-LPC 18:0 | 18:0/0:0 | 5.15±0.25 |
|  |  |  |  | **Total% 1-LPC** | **80.47±0.48** |
|  |  |  |  | **Total% 2-LPC** | **19.53±0.48** |
|  |  |  |  | **Total%** | **100** |
| Values (percent by relative peak area) represent means ± standard errors, n = 4 per experimental replicate. RT = retention time on HILIC chromatography, LPC = lysophosphatidylcholine, LPE = lysophosphatidylethanolamine, LPA = lysophosphatidic acid. The lipid components in the table are arranged based on *m/z* of the molecular species [M-H]^-^ and [M+CH_3_COO]^-^ ions with the number before the colon representing total number of carbons, the numbers after the colon representing the total number of double bonds of the fatty acyl composition (*e.g.*, LPC 18:2 represents PE molecules with a fatty acyl of 18 carbons and 2 double bonds). The position of fatty acyl on glycerol backbone was denote with the order *sn*-1/*sn*-2. | | | | | |


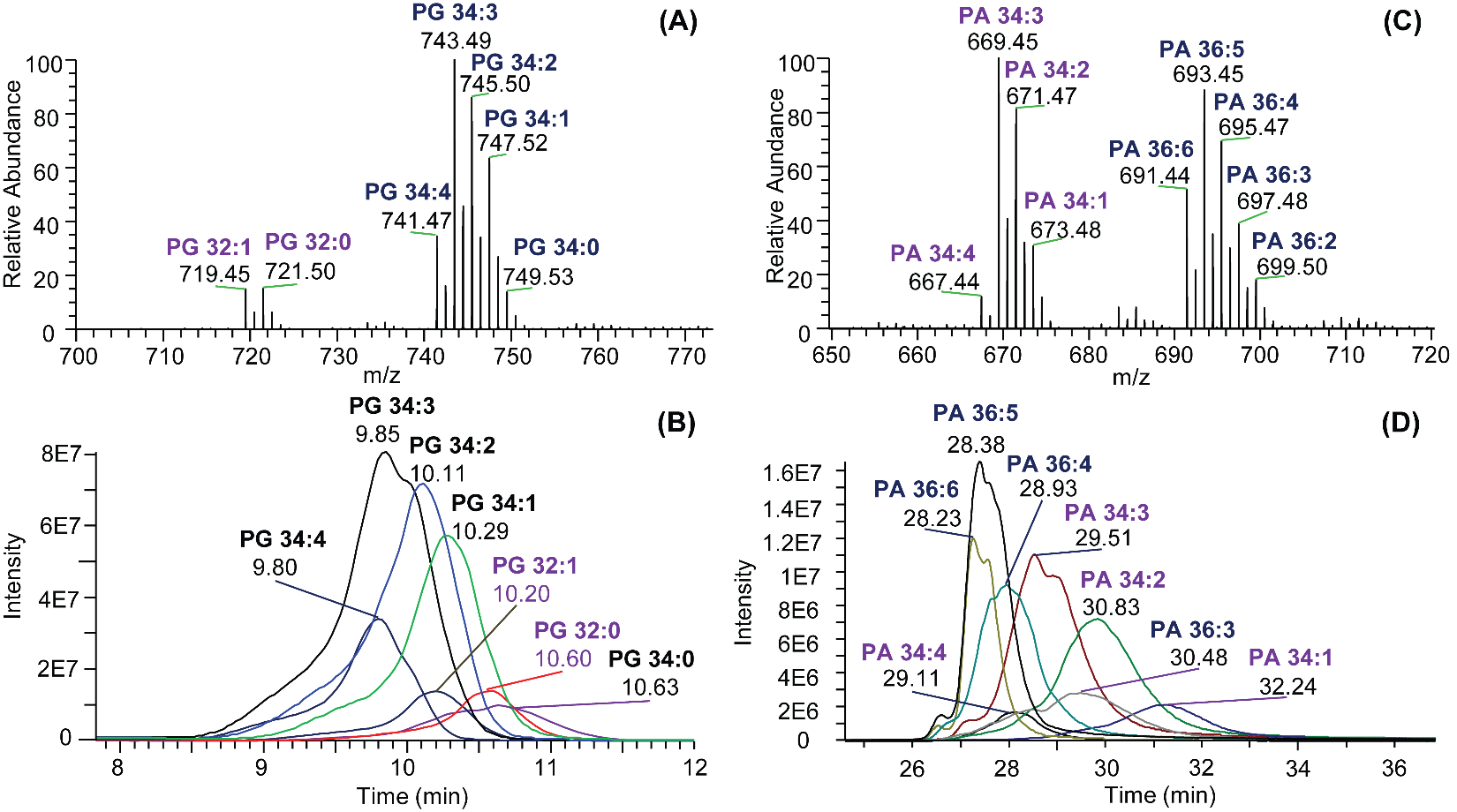


**Figure S-1**. Partial intra-class resolution of phospholipids in plant samples by UHPLC-HILIC-HR/MS. Chromatographic and HR/MS separation of PG (a-b) and PA (c-d) in negative ion mode respectively.


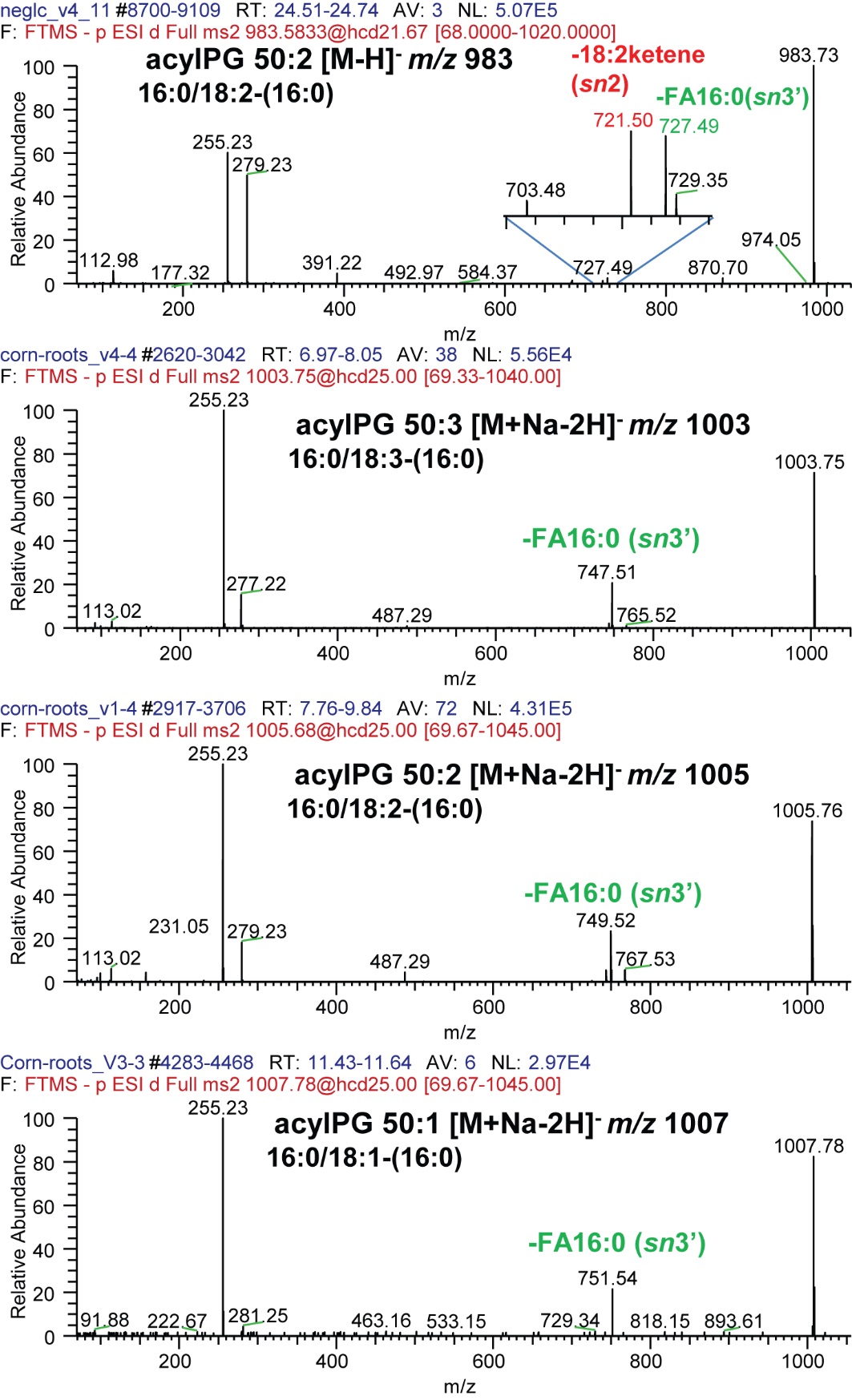


**Figure S-2**. Additional CID spectra from HILIC-MS/MS and C30RP-MS/MS of acylPG 50:1, acylPG 50:2 and acylPG 50:3 lipids present in silage corn roots following cultivation in cool climatic conditions.


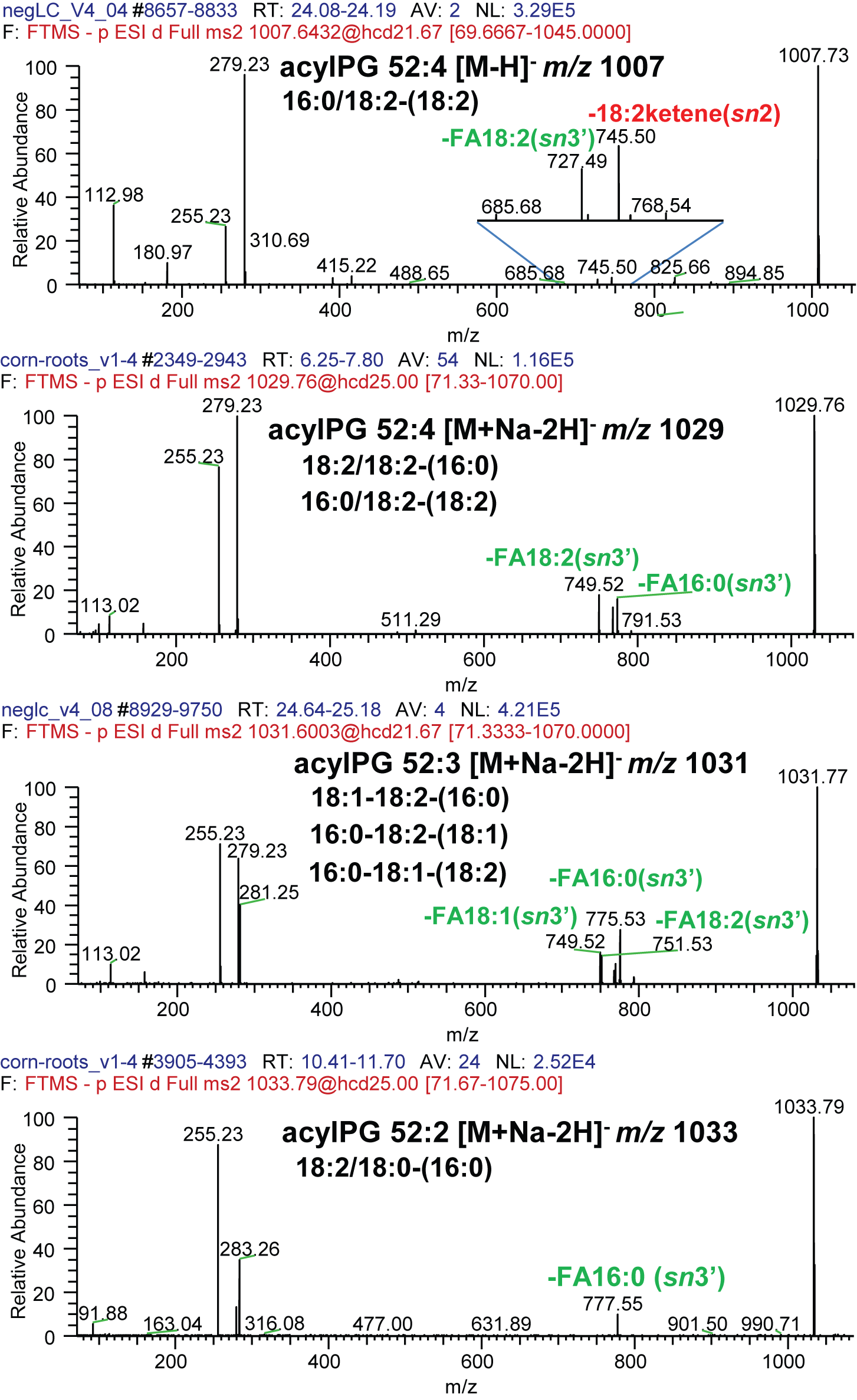


**Figure S-3**. Additional CID spectra from HILIC-MS/MS and C30RP-MS/MS of acylPG 52:2, acylPG 52:3 and acylPG 52:4 lipids present in silage corn roots following cultivation in cool climatic conditions.
